# Genetic Influence Underlying Brain Connectivity Phenotype: A Study on Two Age-Specific Cohorts

**DOI:** 10.3389/fgene.2021.782953

**Published:** 2022-02-07

**Authors:** Shan Cong, Xiaohui Yao, Linhui Xie, Jingwen Yan, Li Shen, and the Alzheimer’s Disease Neuroimaging Initiative

**Affiliations:** ^1^ Department of Biostatistics, Epidemiology and Informatics, Perelman School of Medicine, University of Pennsylvania, Philadelphia, PA, United States; ^2^ Department of Electrical and Computer Engineering, School of Engineering, Indiana University Purdue University Indianapolis, Indianapolis, IN, United States; ^3^ Department of BioHealth Informatics, School of Informatics and Computing, Indiana University Purdue University Indianapolis, Indianapolis, IN, United States

**Keywords:** causal inference, body mass index, genome-wide association study, human connectomics, network segregation

## Abstract

**Background:** Human brain structural connectivity is an important imaging quantitative trait for brain development and aging. Mapping the network connectivity to the phenotypic variation provides fundamental insights in understanding the relationship between detailed brain topological architecture, function, and dysfunction. However, the underlying neurobiological mechanism from gene to brain connectome, and to phenotypic outcomes, and whether this mechanism changes over time, remain unclear.

**Methods:** This study analyzes diffusion-weighted imaging data from two age-specific neuroimaging cohorts, extracts structural connectome topological network measures, performs genome-wide association studies of the measures, and examines the causality of genetic influences on phenotypic outcomes mediated via connectivity measures.

**Results:** Our empirical study has yielded several significant findings: 1) It identified genetic makeup underlying structural connectivity changes in the human brain connectome for both age groups. Specifically, it revealed a novel association between the minor allele (G) of rs7937515 and the decreased network segregation measures of the left middle temporal gyrus across young and elderly adults, indicating a consistent genetic effect on brain connectivity across the lifespan. 2) It revealed rs7937515 as a genetic marker for body mass index in young adults but not in elderly adults. 3) It discovered brain network segregation alterations as a potential neuroimaging biomarker for obesity. 4) It demonstrated the hemispheric asymmetry of structural network organization in genetic association analyses and outcome-relevant studies.

**Discussion:** These imaging genetic findings underlying brain connectome warrant further investigation for exploring their potential influences on brain-related complex diseases, given the significant involvement of altered connectivity in neurological, psychiatric and physical disorders.

## 1 Introduction

Brain structural connectivity is a major organizing principle of the nervous system. Estimating interregional neural connectivity, reconstructing geometric structure of fiber pathways, and mapping the network connectivity to corresponding inter-individual variabilities provide fundamental insights in understanding detailed brain topological architecture, function and dysfunction. A large body of research has been devoted to extracting and investigating macro-scale brain networks from diffusion-weighted imaging (DWI) data (Xie et al., 2018; Jiang et al., 2019; van den Heuvel et al., 2019; Bertolero et al., 2019; Elsheikh et al., 2020), and various behavioral, neurological and neuropsychiatric disorders have been linked to the disrupted brain connectivity (Jiang et al., 2019; van den Heuvel et al., 2019). As structural changes of brain connectivity are phenotypically associated with massive complex traits across different categories, the brain-wide connectome has been extensively studied.

It is worth noting that human brain connectome re-configures its network structure dynamically and adaptively in response to genetic, lifestyle, environmental factors (Cohen and D’Esposito, 2016; Cauda et al., 2018), brain development and aging (Sala-Llonch et al., 2015; Alloza et al., 2018; Varangis et al., 2019). However, the underlying neurobiological mechanism from gene to brain connectome, and to cognitive and behavioral outcomes, and whether this mechanism changes over time, remain unclear. To bridge this gap, we perform a genetic study of brain connectome phenotypes on two different age-specific cohorts: one contains healthy young adults (age: 28.7 ± 3.6), and the other contains elderly participants (age: 73.8 ± 7.0). Our goal is to identify genetic factors affecting brain connectivity and examine their consistency and discrepancy between these two age-specific groups.

Emerging advances in multimodal brain imaging, high throughput genotyping and sequencing techniques provide exciting new opportunities to ultimately improve our understanding of brain structure and neural dynamics, their genetic architecture and their influences on cognition and behavior (Shen and Thompson, 2020). Present studies investigating direct associations among human connectomics, genomics and clinical phenotyping are primarily focused on four aspects: 1) estimating genetic heritability of basic connectome measures such as number of fibers, length of fibers and fractional anisotropy (FA) (Jahanshad et al., 2013; Thompson et al., 2013; Elliott et al., 2018); 2) discovering pairwise univariate associations between single nucleotide polymorphisms (SNPs) and imaging phenotypic traits such as above mentioned basic connectome measures at each edge (Jahanshad et al., 2013; Karwowski et al., 2019) and white matter properties at each voxel (Kochunov et al., 2010; Alloza et al., 2018; Guo et al., 2020); 3) discovering pairwise univariate associations between SNPs and clinical phenotypes such as cognitive or behavioral outcomes (Jahanshad et al., 2013; Elsheikh et al., 2020); and 4) discovering pairwise univariate associations between basic connectome measures and clinical phenotypes (Jiang et al., 2019; van den Heuvel et al., 2019).

Among the studies mentioned above, there exist two major limitations. First, these studies were conducted based on basic connectome measures such as number of fibers, length of fibers and FA, but the complex-network attributes were overlooked, which included network segregation, integration, centrality and resilience and important network components such as hubs, communities, and rich clubs (Sporns, 2013). These attributes were extensively adopted to detect network integration and segregation, quantitatively measure the centrality of network regions and pathways, characterize patterns of local anatomical circuitry, and test resilience of networks to insult (Rubinov and Sporns, 2010). Second, these studies performed analyses by examining the association between an independent variable (e.g., SNP) and a dependent variable (e.g., cognitive or behavioral outcome), without taking into consideration the mediator(s) linking these variables (Baron and Kenny, 1986). Mediation analysis can help identify the underlying mechanism of outcome-relevant genetic effects implicitly mediated by neuroimaging phenotypes (e.g., connectome measures). Of note, mediation analysis requires the independent variable to be significantly associated with both the dependent variable and the mediator. This makes applying it in brain neuroimaging studies a challenge due to the modest effect size of an individual genetic variant on both behavioral and imaging phenotypes (Saykin et al., 2015; Cong et al., 2018), as well as limited size of the sample with all diagnostic, imaging and genetic data available.

With the demand of measuring complex-network attributes, a few recent genome-wide association studies (GWAS) (Bertolero et al., 2019; Elsheikh et al., 2020) recognized the first problem mentioned above and adopted quantitative measurement approaches for complex-network attributes, and treated the attributes as neuroimaging traits for the explorations of complex imaging genomic associations. They successfully identified a number of loci susceptible for Alzheimer’s disease (Elsheikh et al., 2020), and demonstrated the associations between loci and segregated network patterns, which may be involved in brain development, evolution, and disease (Bertolero et al., 2019). However, a notable limitation is that these studies only focus on the brain networks of either young or elderly participants, as a result, their study outcomes are lack of validations in multiple data sets. Since there is an age-related discrepancy for genetic effects on human connectome alterations across lifespan (Varangis et al., 2019), it remains an under-explored topic to examine genetic consistency and discrepancy for complex-network attributes among cohorts different in age. Another factor that may cause discrepancy in the network architecture is the hemispheric asymmetry (Jiang et al., 2019), and the hemispheric asymmetry of network organization has been linked to development processes (Zhong et al., 2017) and neuropsychiatric disorders (Sun et al., 2017). It remains a challenge to understand the genetic basis for the network attributes of two hemispheres as they may be distinctively correlated to cognition level, physical and psychological development.

Among a large number of complex-network attributes, it has been well documented in recent literatures (Cohen and D’Esposito, 2016; Xie et al., 2018) that segregation of neural information such as modularity, transitivity, clustering coefficients and local efficiency represent the connectivity of local network communities that are intrinsically densely connected and strongly coupled. A converging evidence (Cohen and D’Esposito, 2016; Karwowski et al., 2019) is shown that local, within-network communication is critical for motor execution, whereas integrative, between-network communication is critical for measuring connectome (Bertolero et al., 2019). Thus, network segregation is thought to be essential for describing and understanding of complex neural connectome systems (Sporns, 2013). In addition, segregation measures are highly reliable and heritable network attributes (Xie et al., 2018), and these measures have been linked to the disruption of neural network connectivity in brain development, evolution, disease (Cohen and D’Esposito, 2016; Mak et al., 2016; Bertolero et al., 2019), and immunodeficiency (Bell et al., 2018). Given the importance of network segregation, in this study, we first focus on quantifying measures of network segregation, analyzing heritability of segregation measures and performing genetic association analyses by treating them as neuroimaging traits. Then, our next priority is to explore the genetic basis for the rest of the complex-network attributes (e.g. integration, centrality and resilience).

**FIGURE 1:**
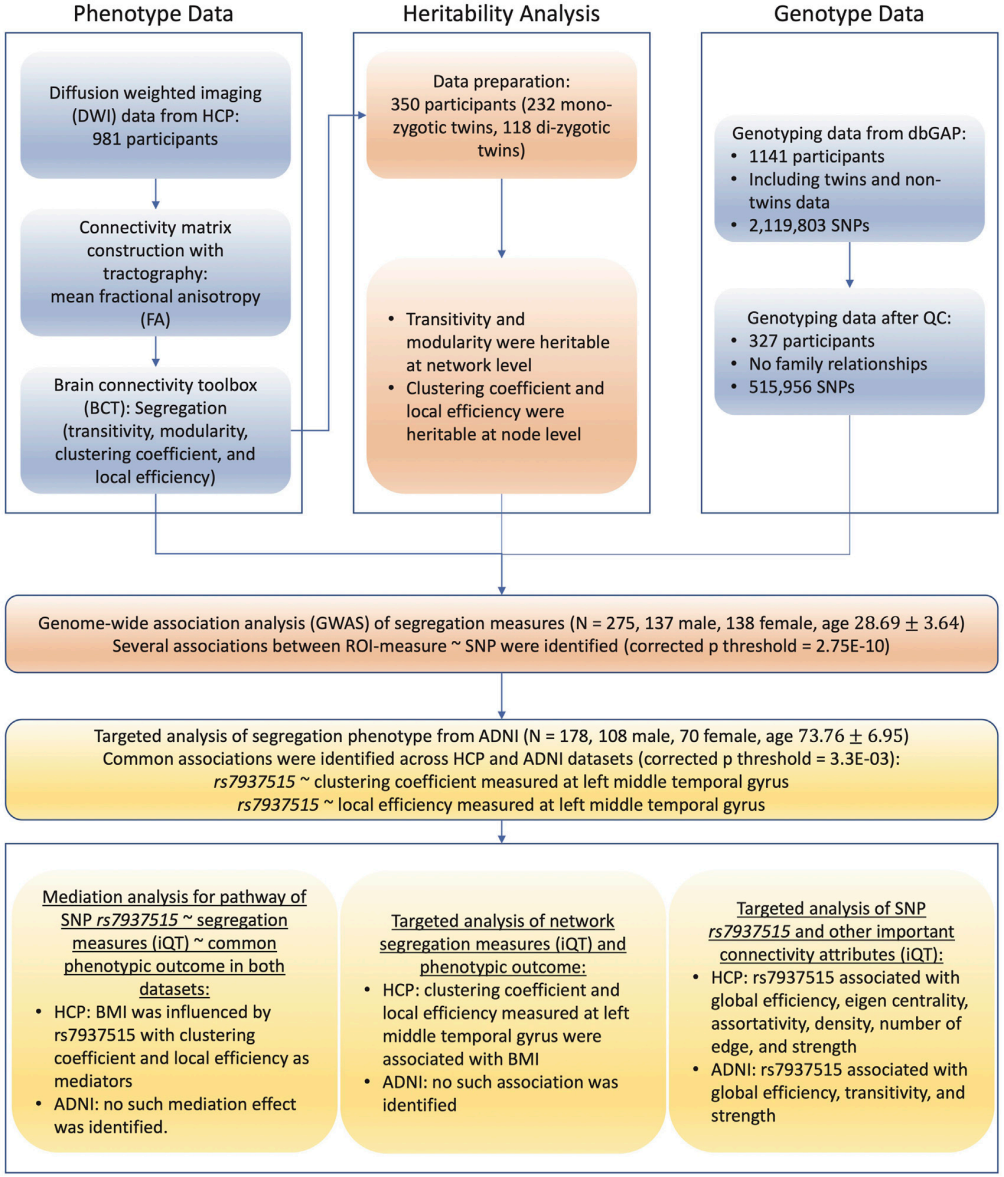
Flowchart of brain connectome GWAS design. Abbreviations: SNPs, single nucleotide polymorphisms; ADNI, Alzheimer’s disease neuroimaging initiative; HCP, human connectome project; dbGaP, database of genotypes and phenotypes; QC, quality control; ROI, region of interest; iQT: imaging quantitative trait; BMI, body mass index.

To overcome the challenges mentioned above, this study aims to develop and implement computational and statistical strategies for a systematic characterization of structural connectome optimized for imaging genetic studies, and to determine genetic basis of structural connectome. Specifically, the framework is organized and described in Figure 1, and the primary goals are to address the following six critical issues: 1) construction of basic network connectivity with diffusion tractography, 2) systematic extraction of complex-network attributes, 3) heritability analysis of complex-network attributes, 4) genome-wide association studies of quantitative endophenotypes, 5) examination of mediation effect that intermediately bridges genes and outcomes, and 6) identification of outcome-relevant neuroimaging biomarkers. Given the enormously broad scope of brain connectome, our focus is on studying 1) static tractography-based structural connectome and complex-network attributes characterizing segregation, integration, centrality and resilience; 2) genetic consistency and discrepancy for complex-network attributes among cohorts different in age; and 3) mediation effects of network attributes on outcome-relevant genetics.

The major contributions of this study are fivefold:

• **New challenges in human connectome:** we elucidate the neurobiological pathway from SNPs to brain connectome, and to phenotypic outcomes. By integrating connectomics and genetics, this study provides new genetic mechanism insights into understanding detailed brain topological architecture, and encoding (or mapping) inter-regional connectivity in the genome.
• **New genetic insights for brain phenotype:** we validate the study outcomes by examining genetic consistency and discrepancy for complex-network attributes between young adult cohort and elderly adult cohort, which illustrates the genetic basis for human connectome in different life stages.
• **Biological findings:** we treat network segregation measures as imaging quantitative traits (iQT), and demonstrate that body mass index [BMI, which is related to multiple complex diseases (Emmerzaal et al., 2015; Stenholm et al., 2017)] is influenced by a locus rs7937515 with network segregation attributes (e.g., clustering coefficient and local efficiency) measured at the left middle temporal gyrus as mediators, which reveals the intermediate effects of brain connectivity in the pathway of outcome-relevant genetics.
• **Biological findings:** we discover network segregation as an important neuroimaging biomarker for BMI and weight-related disorders, and illustrate the importance of the left middle temporal gyrus for BMI.
• **Biological findings:** we demonstrate the hemispheric asymmetry of structural network organization in genetic association analyses and outcome-relevant studies.

## 2 Materials and Methods

### 2.1 Study Datasets

With the purpose of examining genetic consistency and discrepancy for complex-network attributes between young and elderly adults, and illustrating genetic basis for human connectome in different life stages, our analysis was respectively conducted on Human Connectome Project (HCP) database for young adults and Alzheimer’s disease Neuroimaging Initiative (ADNI) database for elderly adults.

#### 2.1.1 HCP Young Adult Dataset

HCP (Van Essen et al., 2013) is a major endeavor to map macroscopic human brain circuits and their relationship to behavior in a large population. It aims to reveal the neural pathways that underlie brain function and behavior, by acquiring and analyzing human brain connectivity from high-quality neuroimaging data in healthy young adults. The HCP datasets serve as a key resource for the neuroscience research community, as it provides valuable resources for characterizing human brain connectivity and function, their relationship to behavior, and their heritability and genetic underpinnings, which enables discoveries of how the brain is wired and how it functions in different individuals.

#### 2.1.2 ADNI Elderly Adult Dataset

Alzheimer’s disease Neuroimaging Initiative (ADNI) database was initially launched in 2004 as a public-private partnership, and led by the Principal Investigator Michael W. Weiner, MD. One primary aim of ADNI has been to examine whether serial imaging biomarkers extracted from MRI, positron emission tomography (PET), other biological markers, and clinical and neuropsychological assessment can be combined to measure the progression of mild cognitive impairment (MCI) and early AD. For up-to-date information, see www.adni-info.org.

### 2.2 Demographics

We initially downloaded 981 subjects from HCP database, including a part of twin subjects, then one individual from each family was randomly selected and excluded. As a result, 275 unrelated participants were selected for further population-based genetic analyses. ADNI data were collected by selecting the participants who had both genotype data and baseline DWI data at their first visit, family relationship was also removed in the same way as described above for HCP data filtration. Detailed characteristic information and the number of subjects in each data cohort are shown in Table 1. In this study, we analyzed a total of 275 participants (age: 28.7 ± 3.6; gender: 137 male, 138 female; education: 15.1 ± 1.6) from the HCP database, and a total of 178 participants (age: 73.8 ± 7.0; gender: 108 male, 70 female; education: 16.0 ± 2.8) from the ADNI database. This study was approved by institutional review boards of all participating institutions, and written informed consent was obtained from all participants or authorized representatives.

**TABLE 1:** Participant characteristics in HCP and ADNI genetic association analyses.

Cohort	HCP	ADNI	*p*
Number	275	178	—
Gender (M/F)	137/138	108/70	**3.02E-02**
Age	28.69 ± 3.64	73.76 ± 6.95	**5.56E-175**
Education	15.14 ± 1.64	16.03 ± 2.78	**1.41E-04**
MMSE	29.09 ± 1.04	27.37 ± 2.54	**2.28E-15**
Weight	77.70 ± 17.06	77.71 ± 15.92	1.00
BMI	25.99 ± 4.73	27.28 ± 5.24	**8.87E-03**
clus coef ROI 087	0.51 ± 0.05	0.29 ± 0.13	**1.41E-55**
loc effi ROI 087	0.52 ± 0.05	0.39 ± 0.17	**1.20E-18**

*p*-values were assessed because of significant differences among diagnosis groups, and were computed using one-way ANOVA (except for gender using *χ* ^2^ test). The *p* values < 0.05 are shown in bold. HC = healthy control; EMCI = early mild cognitive complaint; LMCI = late mild cognitive complaint; AD = Alzheimer’s disease.

### 2.3 Genotyping Data Acquisition and Processing

#### 2.3.1 HCP Young Adults Dataset

HCP samples were genotyped using MEGA array with PsychChip and ImmunoChip content. 1,141 genotype data was downloaded from dbGAP. Quality control was performed in PLINK v1.90 (Purcell et al., 2007) using the following criteria: 1) call rate per marker ≥98% , 2) minor allele frequency (MAF) ≥5% , 3) Hardy Weinberg Equilibrium (HWE) test *p* ≤ 1.0E-6, and 4) call rate per participant ≥98% . Variants with no “rs” number, and samples with evidence of identity-by-descent (IBD) ≥0.25 or heterozygosity rate ±3 standard deviations from the mean were further excluded. Following quality control process, the number of samples with genotype data reduced to 327, we then checked the missing data by matching subjects information between phenotype and genotype data. As a result, this study comprised a total of 327 unrelated subjects and 515,956 SNPs.

#### 2.3.2 ADNI Elderly Adults Dataset

Genotyping data were obtained from the ADNI database (adni.loni.usc.edu). They were quality-controlled as described in (Cong et al., 2020; Yao et al., 2020). We then performed imputation to maximize the number of overlaps between HCP GWAS findings and ADNI SNPs, see (Yao et al., 2019) for details. Briefly, genotyping was performed on all ADNI participants following the manufacturer’s protocol using blood genomic DNA samples and Illumina GWAS arrays (610-Quad, OmniExpress, or HumanOmni2.5-4v1) (Saykin et al., 2010). Quality control was performed in PLINK v1.90 (Purcell et al., 2007) using the following criteria: 1) call rate per marker ≥95% , 2) minor allele frequency (MAF) ≥5% , 3) Hardy Weinberg Equilibrium (HWE) test *p* ≤ 1.0E-6, and 4) call rate per participant ≥95% . In total, 5,574,300 SNPs were included for further targeted genetic association analysis.

### 2.4 Tractography and Network Construction

#### 2.4.1 Tractography

We downloaded high spatial resolution DWI data and genotype data from both HCP and ADNI databases. DWI data from HCP was processed following the MRtrix3 guidelines (Tournier et al., 2012), detailed procedures have been previously reported (Xie et al., 2018) and are briefly described below: 1) generating a tissue-segmented image; 2) estimating the multi-shell multi-tissue response function and performing the multi-shell multi-tissue constrained spherical deconvolution; 3) generating the initial tractogram and applying the successor of Spherical-deconvolution Informed Filtering of Tractograms (SIFT2) methodology (Smith et al., 2015); and 4) mapping the SIFT2 output streamlines onto the MarsBaR automated anatomical labeling (AAL) atlas (Tzourio-Mazoyer et al., 2002) with 90 ROIs to produce the structural connectome with edge value equal to the mean fractional anisotropy (FA).

DWI data from ADNI was acquired following the scanning protocols described in (Elsheikh et al., 2020), and processed following the procedures discussed in (Yan et al., 2018). Tractography was performed in Camino (Cook et al., 2006) based on white matter fiber orientation distribution function (ODF). As Camino adopted a deterministic approach, streamlines were modeled with a multi-tensor modeling approach (voxels fitted up to three fiber orientations, this way accounting for most of the fiber-crossings) of the ODF data. To generate a comparable tractography, the streamlines were also mapped onto AAL atlas with 90 ROIs to produce the structural connectome with edge value equal to the mean FA.

#### 2.4.2 Network Construction

Network was created and defined by connectivity matrix *M* where *M*_*ij*_ stores the connectivity measure between ROIs *i* and *j*. As described in the previous section, we considered FA for defining *M*_*ij*_. Since the diffusion tensor is a symmetric 3 × 3 matrix, it can be described by its eigenvalues (*λ*_1_, *λ*_2_ and *λ*_3_) and eigenvectors (*v*_1_, *v*_2_ and *v*_3_) for tractography analysis. FA at edge-level is an index for the amount of diffusion asymmetry within a voxel, defined in terms of its eigenvalues:

$$FA=\sqrt{\frac{{\left(\lambda_{1}-\lambda_{2}\right)}^{2}+{\left(\lambda_{2}-\lambda_{3}\right)}^{2}+{\left(\lambda_{1}-\lambda_{3}\right)}^{2}}{2\left(\lambda_{1}^{2}+\lambda_{2}^{2}+\lambda_{3}^{2}\right)}}.$$ (1)

Thus, mean FA is a normalized measure of the fraction of the tensor’s magnitude due to anisotropic diffusion, corresponding to the degree of anisotropic diffusion or directionality.

### 2.5 Complex-Network Attributes

With an undirected and weighted connectivity matrix *M* (defined in Section 2.4.2), we assessed a comprehensive set of network features such as segregation (e.g., transitivity, clustering coefficients, local efficiency and modularity), integration (e.g., global efficiency), centrality (e.g., eigen centrality) and resilience (e.g., assortativity) of the structural connectome using Brain Connectivity Toolbox (BCT) (Rubinov and Sporns, 2010). Given the importance and priority of segregation measures in this study, we only introduced the definitions of segregation measures, and the definitions of the rest complex-network attributes were explained in (Rubinov and Sporns, 2010).

For the following sub-sections, we define *N* as the set of all nodes in the network, *n* as the number of nodes, *t*_*i*_ as geometric mean of triangles around node $i\;\left(t_{i}=\frac{1}{2}\sum_{j,h\in N}{\left(M_{ij}M_{ih}M_{jh}\right)}^{1/3}\right)$ , *k*_*i*_ as weighted degree of *i* (*k*_*i*_ = *∑*_*j*∈*N*_ *M*_*ij*_), *a*_*ij*_ as the connection status between *i* and $j\;\left(\right.\;a_{ij}=1$ when link (*i*, *j*) exists, *a*_*ij*_ = 0 otherwise), *d*_*ij*_ as shortest weighted path length between *i* and $j(\;\left(\right.\;d_{ij}=\sum_{a_{uv}\in g_{i↪j}}f\left(M_{uv}\right)$ , where *f* is a map from weight to length and *g*_*i*↪*j*_ is the shortest weighted path between *i* and *j*).

#### 2.5.1 Transitivity

Transitivity measures the ratio of triangles to triplets in the network. By following the definition in (Newman, 2003):

$$T=\frac{\sum_{i\in N}2t_{i}}{\sum_{i\in N}k_{i}\left(k_{i}-1\right)},$$ (2)

where *T* is the transitivity measured at network level.

#### 2.5.2 Clustering Coefficient

Clustering coefficient measures the degree to which nodes in a network tend to cluster together by evaluating the fraction of triangles around a node and is equivalent to the fraction of node’s neighbors that are neighbors of each other. By following the definition in (Onnela et al., 2005):

$$C=\frac{1}{n}\underset{i\in N}{\sum }C_{i}=\frac{1}{n}\underset{i\in N}{\sum }\frac{2t_{i}}{k_{i}\left(k_{i}-1\right)},$$ (3)

where *C*_*i*_ is the clustering coefficient of node *i* and *C* is the clustering coefficient measured at network level.

#### 2.5.3 Local Efficiency

Local efficiency measures the efficiency of information transfer limited to neighboring nodes by evaluating the global efficiency computed on node neighborhoods. By following the definition in (Latora and Marchiori, 2001):

$$E_{\text{loc}}=\frac{1}{n}\underset{i\in N}{\sum }\frac{\sum_{j,h\in N,j\neq i}{\left(M_{ij}M_{ih}{\left[d_{jh}\left(N_{i}\right)\right]}^{-1}\right)}^{1/3}}{k_{i}\left(k_{i}-1\right)},$$ (4)

where *E*_loc_ is the local efficiency of node *i*, and $d_{jh}\left(N_{i}\right)$ is the length of the shortest path between *j* and *h*, that contains only neighbors of *i*.

#### 2.5.4 Modularity

Modularity measures network segregation into distinct networks, and it is a statistic that quantifies the degree to which the network may be subdivided into such clearly delineated groups (Newman, 2006):

$$Q=\frac{1}{l}\underset{i,j\in N}{\sum }\left[M_{ij}-\frac{k_{i}k_{j}}{l}\right]\delta_{m_{i},m_{j}},$$ (5)

where *Q* is the modularity measured at network level, *m*_*i*_ is the module containing node *i*, and $\delta_{m_{i},m_{j}}=1$ if *m*_*i*_ = *m*_*j*_, and 0 otherwise.

### 2.6 Heritability Analysis

Heritability analysis focused on identifying highly heritable measures of structural brain networks, and it was a commonly adopted and critical measurement to describe properties of the inheritance of iQT. An iQT such as network attributes must be heritable, which was defined as the proportion of phenotypic variance due to genetic differences between individuals (Jørstad and Næevdal, 1996). In this study, we estimated heritability of four segregation measures from twin subjects in the HCP young adult cohort (*N* = 350, 232 mono-zygotic twins, 118 di-zygotic twins) and SOLAR-Eclipse software (Kochunov et al., 2015) was employed for this task. The inputs to this software included phenotype traits, covariates measures and a kinship matrix indicating the pairwise relationship between twin individuals. A maximum likelihood variance decomposition method was applied to estimate the variance explained by additive genetic factors and environmental factors respectively. The output from SOLAR-Eclipse included heritability (h2), standard error and the corresponding significance *p*-value for each feature. We estimated the heritability of connectomic features, including transitivity, clustering coefficients, local efficiency and modularity. Since many previous studies had reported the effect of age (linear nonlinear), gender and their interactions on structural brain connectivity (Burzynska et al., 2010; Gong et al., 2011; Lopez-Larson et al., 2011; Zhao et al., 2015), all heritability analyses were performed with age, age^2^, sex, age×sex and age^2^×sex as covariates. In addition, we extracted the total variance explained by all covariate variables.

### 2.7 Brain Connectome Genetic Association Analysis

#### 2.7.1 HCP Cohort

GWAS on the brain network segregation measures of the 90 ROIs were performed using linear regression under an additive genetic model in PLINK v1.90 (Purcell et al., 2007). Age, gender and education were included as covariates. Our GWAS was focused on analyzing the following network segregation measures: 1) modularity and transitivity, which were network-level measures; and 2) clustering coefficient and local efficiency, which were node-level measures. As a result, in total, we have 2 + 90 × 2 = 182 measures. Our post-hoc analysis used Bonferroni correction for correcting the genome-wide significance (GWS) for the number of quantitative traits (i.e., 5E-8/182 = 2.75E-10).

#### 2.7.2 ADNI Cohort

Genetic findings of the segregation measures from HCP young adult dataset were treated as genotypic candidates and segregation measures at specific ROIs as phenotypic candidates, we further examined in ADNI elderly adult dataset regarding their associations. Apart from including age, gender and education as covariates, we also added diagnostic status into the linear regression model, as a large part of ADNI participants suffered from cognitive disorders. By validating the genetic findings from HCP data using ADNI participants, we examined genetic consistency and discrepancy for network segregation attributes between young and elderly adults, which illustrated the consistency and discrepancy of genetic basis for human connectome in different life stages.

In addition, the validated genetic findings were used to further explore connectivity variances with all important complex-network attributes excepting segregation measures such as integration (e.g., global efficiency and network density), centrality (e.g., eigen centrality) and resilience (e.g., assortativity), and we examined the targeted genetic basis on certain brain ROIs (e.g., middle temporal gyrus). As previously stated, linear regression models were used. In particular, we applied additive genetic models implemented in PLINK v1.90, with age, gender, education as covariates.

### 2.8 Mediation Analysis

To examine the causal assumption, we followed the Baron-Kenny procedure (Baron and Kenny, 1986) to perform standard mediation analysis to identify the mediated effect, and we treated iQTs (e.g., network segregation measures) as mediating variables, which intermediately linked the pathological path from genetic findings to clinical phenotypes. Specifically, we constructed a set of candidate SNPs which were found significantly associated to segregation measures in both young and elderly participants, and we constructed a set of candidate clinical phenotyping information by extracting overlapped clinical outcomes collected in both HCP and ADNI databases. We then employed the mediation model to detect the indirect effect of loci on clinical outcomes via iQT.

Specifically, mediation analysis was performed using the non-parametric bootstrap approximation with the R “mediation” package developed by Imai et al. (2010). Let *y* be the dependent variable which was a clinical outcome in our study, *x* be the independent variable which was a candidate SNP, *z* be the covariates (age, gender and education), and *M* be the mediating variable which was brain iQT. The mediation analysis was conducted in 3 steps:

1) fit a linear model to regress the mediating variable *M* against SNP *x* while controlling for *z*;
2) fit a linear model to regress the clinical outcome *y* against SNP *x* while controlling for *z*;
3) adopted the non-parametric bootstrap approximation to estimate the direct effect, mediation effect, proportion of total effect via mediation, their 95% confidence intervals (CI) and p values, by conducting 1,000 simulations.

### 2.9 Outcome-Relavent Brain Connectome Association Analysis

To discover the outcome-relevant biomarkers which mapped brain connectivity alterations to cognitive or behavioral outcomes, we performed pairwise univariate association analysis between network segregation attributes and outcome data. We selected BMI and Mini-Mental State Examination (MMSE) as outcomes as they were not only measures available in both HCP and ADNI cohorts but also important quantitative traits related to complex diseases such as weight-related disorders as well as neurological and psychiatric disorders. We used linear regression to regress the phenotypic outcomes against network segregation measures for both HCP and ADNI datasets, and explored outcome-relevant brain neuroimaging biomarkers. By comparing young and elderly participants, we illustrated the consistency and discrepancy of human brain connectome in different ages regarding on BMI and MMSE variations.

## 3 Results

### 3.1 Heritability of Network Segregation

As illustrated in Figure 1, we examined segregation measures estimated at both network-level and node-level prior to GWAS. All of the segregation measures such as clustering coefficients (node-level), local efficiencies (node-level), transitivity (network-level) and modularity (network-level) showed significantly high heritability after Bonferroni correction (p <0.05/182=2.75E-04 ). The mean (±std) heritability of 182 segregation measures (h2 score) was 0.81 (±0.05), and more detailed results of heritability analysis were listed in Supplementary Table. We included all 182 segregation measures in the subsequent GWAS analysis.

### 3.2 GWAS of Network Segregation in HCP Young Adults

**FIGURE 2:**
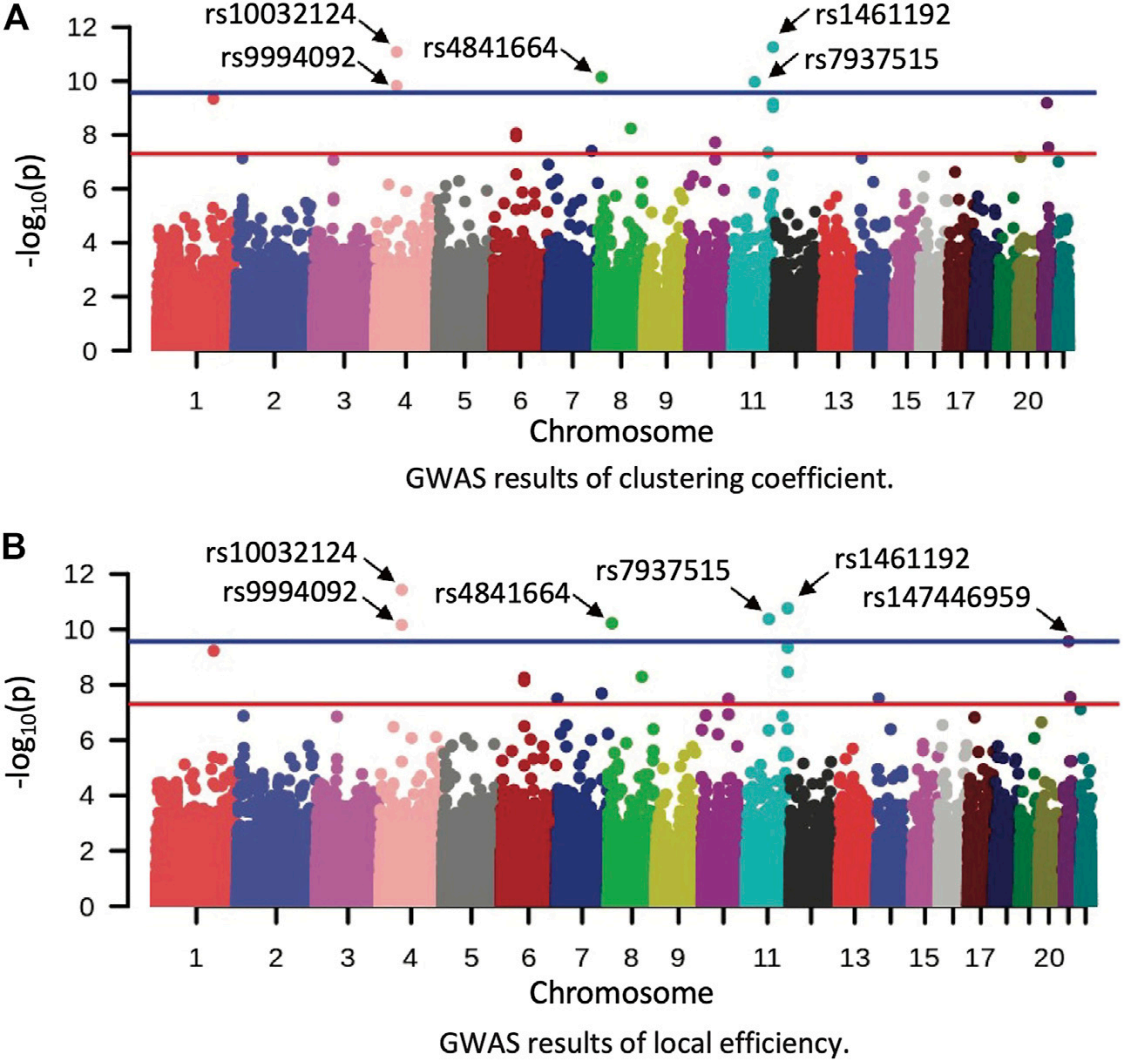
Manhattan plot of GWAS results in the HCP dataset. **(A,B)** show the GWAS results of clustering coefficient and local efficiency on left middle temporal gyrus, respectively. Red and blue lines correspond to the *p*-value of 5E-08 and 2.75E-10, respectively.

In the HCP cohort, genome-wide associations between 515, 956 SNPs and 182 structural network segregation measures were assessed under the additive genetic model and controlled for age, gender and education. GWAS identified 20 significant associations between 10 SNPs and 7 segregation measures (Table 2), after correcting the genome-wide significance (GWS) for the number of phenotypes using Bonferroni method (i.e., *p* <5E-08/182=2.75E-10 ). Respectively shown in Figure 2 were Manhattan plots of GWAS results of clustering coefficient and local efficiency measured in left middle temporal gyrus. GWAS of HCP data showed high consistency for clustering coefficient and local efficiency, where nine SNP-ROI associations were discovered for these two segregation measures. After Bonferroni correction, there was no significant finding for the network level segregation measures (i.e., transitivity and modularity).

**TABLE 2:** Significant associations between SNPs and segregation measures: statistics in the HCP and ADNI cohorts.

Segregation measure	ROI	CHR	SNP	BP^a^	Closest gene^b^	HCP		ADNI	
						Beta	*p*	Beta	P^c^
Clustering coefficient	FMidO_R	6	rs6930337	148788006	—	−0.36	**1.25E-10**	−0.10	1.65E-01
		18	rs1940608	5927441	TMEM200C	−0.39	**8.00E-12**	0.09	2.15E-01
		18	rs4798416	5930979	TMEM200C	−0.39	**7.52E-12**	0.09	2.15E-01
	FMedO_R	10	rs2104994	5273767	AKR1C4	−0.37	**7.71E-11**	−0.10	1.98E-01
	TPMid_L	4	rs9994092	66 436 114	—	−0.37	**1.51E-10**	−0.05	4.71E-01
		4	rs10032124	66 485 112	—	−0.39	**8.26E-12**	−0.04	5.68E-01
		8	rs4841664	11 859 985	DEFB134/DEFB135/	−0.38	**7.16E-11**	—	—
					DEFB136				
		11	rs7937515	71 841 325	ANAPC15/LRTOMT/	−0.37	**1.09E-10**	−3.20	**1.63E-03**
					FOLR3/LAMTOR1				
		11	rs1461192	130043580	ST14	−0.40	**5.54E-12**	—	—
Local efficiency	FMidO_R	6	rs6930337	148788006	—	−0.38	**4.68E-11**	−0.07	3.51E-01
		18	rs1940608	5927441	TMEM200C	−0.39	**7.75E-12**	0.10	2.02E-01
		18	rs4798416	5930979	TMEM200C	−0.39	**7.36E-12**	0.10	2.02E-01
	FMedO_L	10	rs2104994	5273767	AKR1C4	−0.37	**1.56E-10**	−0.11	1.33E-01
	FMedO_R	10	rs2104994	5273767	AKR1C4	−0.38	**1.86E-11**	−0.11	1.62E-01
	TPMid_L	4	rs9994092	66 436 114	—	−0.38	**6.84E-11**	−0.05	4.82E-01
		4	rs10032124	66 485 112	—	−0.40	**3.71E-12**	−0.04	5.71E-01
		8	rs4841664	11 859 985	DEFB134/DEFB135/	−0.38	**5.98E-11**	—	—
					DEFB136				
		11	rs7937515	71 841 325	ANAPC15/LRTOMT/	−0.38	**4.22E-11**	−0.24	**1.34E-03**
					FOLR3/LAMTOR1				
		11	rs1461192	130043 580	ST14	−0.39	**1.74E-11**	—	—
		21	rs147446959	29 291 173	—	−0.37	**2.72E-10**	-	—

a Build 37, assembly hg19.

b Genes located ±100 kb of the top SNP.

c *p* value reaching the Bonferroni corrected threshold (0.05/20 = 2.25E-03) is shown in bold.

Abbreviations: F = frontal, TP = temporal pole, Mid = middle, Med = medial, O = orbital, L = left, R = right.

### 3.3 Targeted Genetic Association of Segregation in ADNI Elderly Adults

Given the list of significant findings from the aforementioned GWAS of HCP segregation measures, we further examined their statistical significance in the ADNI cohort to identify brain network relevant genetic variants which were consistent for brain aging. We assessed the associations of 15 out of 20 HCP GWAS findings in ADNI cohort, as three SNPs (rs4841664, rs1461192 and rs147446959 are corresponding to 5 associations in Table 2) were not included in ADNI genotyping data. Associations of rs7937515 with clustering coefficient and local efficiency measured in left middle temporal gyrus were duplicated and validated in ADNI cohort with *p* values of 1.63E-03 and 1.34E-03, respectively, where the Bonferroni corrected significant level *p* <0.05/15=3.33E-03 was applied (Table 2).

**FIGURE 3:**
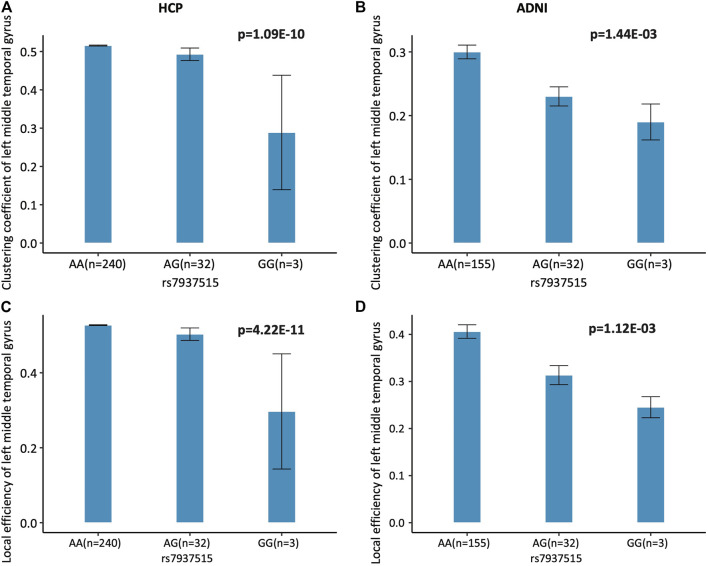
Association of rs7937515 on clustering coefficient and local efficiency of the left middle temporal gyrus. **(A,B)** Mean clustering coefficient with standard errors are plotted against the rs7937515 genotype groups (i.e., AA, AG and GG) for the HCP and ADNI cohorts, respectively. **(C,D)** Mean local efficiency with standard errors are plotted against the rs7937515 genotype groups (i.e., AA, AG and GG) for the HCP and ADNI cohorts, respectively. *p* values are calculated from GWAS (HCP) and targeted analysis (ADNI), and significant *p* values are marked in bold.

The minor allele G of rs7937515 (rs7937515-G) was associated with lower level of both clustering coefficient and local efficiency, compared to its major allele A (Figure 3). We will discuss the risk effect of rs7937515-G on brain function and dysfunction in the discussion section.

### 3.4 Mediation Analysis

According to the genetic association results from the HCP and ADNI subjects, we identified a common genetic finding SNP rs7937515, which was associated with two segregation measures in left middle temporal gyrus (e.g., clustering coefficient and local efficiency). In addition, we extracted two common behavioral and cognitive outcome measures (e.g., BMI and MMSE) by comparing the outcome evaluation methods across the HCP and ADNI databases. Thus, in this section, we had two major focuses: 1) exploring the genetic effect of SNP rs7937515 on outcomes including BMI and MMSE, and gaining deeper insights to the molecular mechanisms of the identified genetic variant, and 2) examining the mediated effect of iQTs (e.g., segregation measures) and illustrating their implicit effects in Eq. 1.

To achieve those two goals, mediation analysis of outcome was performed for evaluating both the direct and implicit effects of rs7937515 on outcomes (i.e., BMI and MMSE) through segregation measurements in left middle temporal gyrus. Mediation analysis required the independent variable (i.e., rs7937515) to be significantly associated with both the dependent variable (i.e., BMI or MMSE) and the mediator (i.e., segregation measurements). Below we respectively reported the mediation results analyzed from both HCP and ADNI data.

**FIGURE 4:**
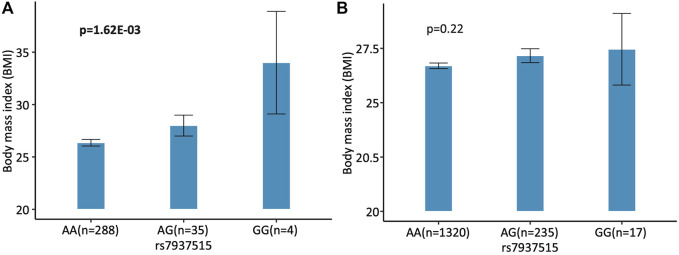
Association of rs7937515 on BMI in the HCP and ADNI cohorts. **(A)** Mean BMI with standard errors are plotted against the rs7937515 genotype groups (i.e., AA, AG and GG) for the HCP cohort. **(B)** Mean BMI with standard errors are plotted against the rs7937515 genotype groups (i.e., AA, AG and GG) for the ADNI cohort. *p* values are calculated from mediation analysis, and significant *p* values are marked in bold.

**FIGURE 5:**
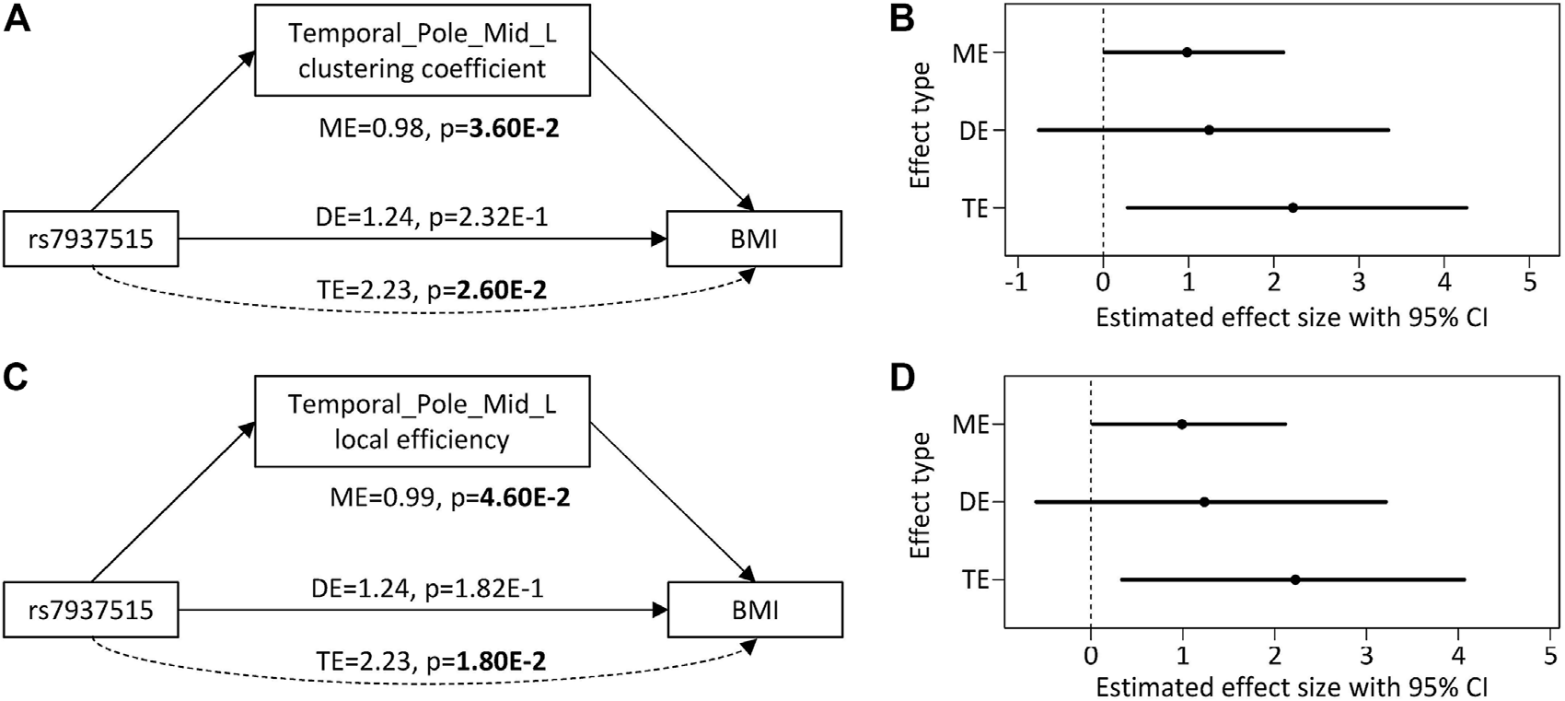
Direct and mediation effect of rs7937515 on BMI through left middle temporal gyrus. **(A,B)** illustrate the effect size, 95% CI and *p* value from rs7937515 mediation analysis of BMI via left middle temporal clustering coefficient. **(C,D)** illustrate the effect size, 95% confidence interval and *p* value from rs7937515 mediation analysis of BMI via left middle temporal local efficient. TE = total effect; DE = direct effect; ME = mediation effect; CI = confidence interval.

For the first focus, the minor allele G of rs7937515 was significantly associated with the increased BMI in HCP cohort (*p* = 1.62E-03; Figure 4A). The same increasing trend was also observed from the ADNI data, although the association between rs7937515 and BMI was not significant (*p* = 0.22; Figure 4B). For the second focus, Figure 5 illustrated the results of mediation analysis with BMI as an outcome measure, from which both clustering coefficient and local efficiency of the left middle temporal gyrus demonstrated the significant intermediate roles between rs7937515 and BMI, with mediation effects of 0.98 (95%CI = [0.06, 2.29], *p* = 3.60E-02) and 0.99 (95%CI = [0.02, 2.11], *p* = 4.60E-02), respectively. There was no significant association between rs7937515 with MMSE in the HCP young adult dataset, so no mediation analysis regarding MMSE was performed. In the ADNI elderly adult dataset, there were no significant associations observed between rs7937515 with BMI nor MMSE; therefore it was not necessary to further examine mediation effects.

Since the brain can be viewed as a predictor, a mediator, or outcome of a health condition (e.g., obesity) (Lowe et al., 2019), it is unclear whether the brain regulates the condition (e.g., structural connectome alteration considered as a mediator for a physical condition such as BMI), or, conversely, brain is affected by the condition. For completeness, we also explored the potential reciprocal relationship from the other direction. The above experiment was repeated with BMI as a mediator and connectivity measures as outcomes. No significant findings were identified, and thus no evidence was observed for BMI as a significant mediator between gene and brain connectivity.

### 3.5 Outcome-Relevant Neuroimaging Biomaker Discoveries

On one hand, for the HCP cohort, we respectively identified significantly negative associations (*p* <0.05/4=1.25E-02 ) between BMI with clustering coefficient (*p* = 3.92E-05) and local efficiency (*p* = 4.57E-05) measured in left middle temporal gyrus. On the other hand, for the ADNI cohort, we examined the associations between BMI and the above mentioned segregation measures in a pair-wise manner, but there was no significant findings satisfying the corrected p threshold. Regarding the relationship between cognitive score (e.g., MMSE) and network segregation measures, there was no significant associations identified for both HCP and ADNI cohorts.

### 3.6 Targeted Genetic Association of Other Important Network Attributes in the Left Middle Temporal Gyrus

To review the genetic effect of SNP rs7937515 from different aspects of network connectivity attributes of the left middle temporal gyrus, we assessed the relationship between rs7937515 and additional node-level measures on reported brain ROI (i.e., left middle temporal gyrus) as well as network-level measures in both HCP and ADNI datasets. Table 3 showed association statistics of rs7937515 with segregation, integration, centrality and resilience measures. After correcting for the number of examined network measures (i.e., *p* <0.05/9=5.56e-03 ), both HCP and ADNI identified significant associations between the targeted SNP with global efficiency (integration) and transitivity (resilience), together with our previous finding that rs7937515 was associated with segregation measures such as clustering coefficient and local efficiency, our results showed the consistent genetic effect of rs7937515 on brain structural network segregation, integration and resilience across aging. Besides the common findings between young and elderly adults, rs7937515 was associated with several other node-level and network-level attributes including network density (integration) and eigenvector centrality (centrality) in HCP data, but not in ADNI. Our results suggested the possible genetic discrepancy for certain brain connectivities in different life stages.

**TABLE 3:** Associations between rs7937515 and brain network measures.

Class	QT	ROI or Global	HCP		ADNI	
			Beta	*p*	Beta	*p*
Segregation	Clustering coefficient	TPMid_L	−0.37	**1.09E-10**	−0.24	**1.63E-03**
	Clustering coefficient	TPMid_R	−0.20	**3.53E-04**	−0.22	**3.94E-03**
	Local efficiency	TPMid_L	−0.38	**4.22E-11**	−0.24	**1.34E-03**
	Local efficiency	TPMid_R	−0.22	**7.05E-05**	−0.22	**2.92E-03**
	Transitivity	Global	−0.23	**3.65E-05**	−0.24	**1.17E-03**
	Modularity	Global	0.20	**5.32E-04**	−0.12	9.32E-02
Integration	Global efficiency	Global	−0.29	**1.63E-07**	−0.24	**1.48E-03**
	Density	Global	−0.26	**2.64E-06**	0.03	7.11E-01
Centrality	Betweenness centrality	TPMid_L	−0.09	1.28E-01	−0.05	5.28E-01
	Betweenness centrality	TPMid_R	−0.06	3.24E-01	−0.03	6.75E-01
	Eigenvector centrality	TPMid_L	−0.32	**9.58E-08**	−0.13	7.85E-02
	Eigenvector centrality	TPMid_R	−0.20	**6.11E-04**	−0.03	6.98E-01
Resilience	Assortativity coefficient	Global	0.10	1.14E-01	0.06	3.95E-01

Abbreviations: TP = temporal pole, Mid = middle, L = left, R = right, QT, quantitative trait. p values reaching the Bonferroni corrected threshold (0.05/9 = 5.56E-03) are shown in bold.

### 3.7 Hemispheric Asymmetry of Brain Connectome

In this study, we noticed a hemispheric asymmetry of outcome-relevant brain connectivity alterations in the left and right middle temporal gyrus (Table 3). Due to two brain regions (e.g., left and right MTG), two segregation measures (e.g., clustering coefficient and local efficiency) and one outcome measure (e.g., BMI), we applied a Bonferroni corrected p threshold in this section (*p* <5E-02/8=6.25E-03 ). In the HCP young adult cohort, for the left MTG, we respectively identified significant associations of BMI with clustering coefficient (*p* = 3.92E-05), and with local efficiency (*p* = 4.57E-05); for the right MTG, even though there were no significant associations of BMI with clustering coefficient (*p* = 2.24E-02), and with local efficiency (*p* = 2.90E-02), both clustering coefficient and local efficiency in left and right MTG showed negative associations with BMI. In the ADNI cohort, as reported in the previous section, network segregation was not associated with BMI, so it was not necessary and proper to conduct analyses regarding ADNI data in this section.

## 4 Discussion

As summarized in Figure 1, prior to GWAS, we first performed heritability analysis for network attributes screening, and only heritable measures of network segregation were treated as iQT for GWAS. Based on experimental outcomes, all of the segregation measures were highly heritable: transitivity and modularity were heritable at network level, clustering coefficient and local efficiency were heritable at all nodes, which suggested segregation measures were suitable for genetic analyses. Then, we performed GWAS of segregation attributes in 275 HCP subjects, and identified several pairwise associations between SNPs and iQTs as listed in Table 2. These GWAS findings were validated in 178 ADNI subjects. As a validation result, we identified several genetic consistency and discrepancy patterns for human connectome in different life stages (as shown in Table 2). As common findings in both HCP young adult and ADNI elderly adult cohorts, rs7937515 was negatively associated with clustering coefficient and local efficiency respectively measured at left middle temporal gyrus. To the best of our knowledge, this was among the first GWAS of human brain high-level network measures across both young and elderly participants. As shown in Figures 3A,C, the minor allele G of rs7937515 was associated with decreased clustering coefficient and local efficiency of the left middle temporal gyrus in both young and elderly participants. As concluded in (Rudie et al., 2013; Keown et al., 2017; Karwowski et al., 2019; Varangis et al., 2019), the weakness of segregated network connectivity (e.g., modularity, clustering coefficient, and local efficiency) was linked to multiple brain disorders such as age-related cognitive declines and autism spectrum disorder. Thus, our GWAS findings for HCP young adults demonstrated that rs7937515 played a risk effect on human network segregation. This neurorisk effect was also confirmed in a targeted genetic association analysis for ADNI elderly participants (as shown in Figures 3B,D), these common discoveries between HCP and ADNI datasets suggested a consistent genetic risk effect across young and old life stages.

This study was further conducted by performing several post-hoc analyses in the following three aspects (shown as bottom sections in Figure 1): 1) examining genetic mechanisms for common outcome measures in the HCP and ADNI studies, and elucidating the mediated effect of iQTs for such outcome-relevant genetic association, 2) discovering outcome-relevant imaging biomarkers, and 3) exploring the genetic mechanisms of other important complex-network attributes.

For the first aspect, our goal was to elucidate the neurobiological pathway from SNPs to brain connectome, and to phenotypic outcome. In addition, we aimed to discover the role of iQTs in the outcome-relevant genetic associations by performing mediation analyses in both HCP and ADNI datasets. For the HCP young participants, we identified that BMI was positively associated with rs7937515 in the first step of mediation analysis, demonstrating a risk effect. rs7937515 located in the regions of *FAM86C1*/FOLR3 was previously discussed in literatures (Gao et al., 2015; Gao, 2017) and positively linked to waist circumference in the meta-analysis based on the Insulin Resistance Atherosclerosis Family Study (IRASFS) (Palmer et al., 2015), which was designed to investigate the genetic and environmental basis of insulin resistance and adiposity. *FAM86C1* (Family With Sequence Similarity 86 Member C1) and *FOLR3* (Folate Receptor Gamma) had been reported for their associations with various weight-related phenotypes such as bone mineral density (Li et al., 2019) and BMI (Hair, 2014; Mrozikiewicz et al., 2019), which closely related to osteoporosis (Li et al., 2019; Mrozikiewicz et al., 2019) and obesity (Gómez-Ambrosi et al., 2004). In the second and third steps of mediation analysis, we illustrated that BMI was indirectly influenced by rs7937515 (Figures 4, 5), and iQTs such as clustering coefficient and local efficiency measured at the left middle temporal gyrus respectively played a mediating role. We also examined the genetic association with MMSE, but no evidence indicated any genetic associations to MMSE. In contrary, for the ADNI elderly participants, neither significant associations between rs7937515 and BMI nor MMSE were identified in the first step of mediation analysis, so there was not a necessary to examine mediated effect in this dataset. Our results demonstrated a disappearance of outcome-relevant genetic effect in the elderly participants, this discrepancy from young to elderly participants might due to the dominated influences from life style, environment or other non-genetic factors.

For the second aspect, recent studies (Lowe et al., 2019; Azevedo et al., 2019) showed that structural changes in brain tissues could affect food consumption behaviors and mediate BMI, which implied connectome alteration could be a causal agent and a promising imaging biomarker in this study. Thus, our goal was set to reveal the mapping between connectivity alterations and phenotypic outcome, and discover outcome-relevant imaging biomarkers. For young adult participants, segregation measures (e.g., clustering coefficient or local efficiency measured at left middle temporal gyrus) previously demonstrated their potential to play a mediating role in genetic association discoveries, in this step, we focused on examining their direct associations to the outcomes. Thus, we performed a targeted association analysis between the mentioned segregation measures and the common outcomes (e.g., BMI or MMSE) evaluated in both HCP and ADNI studies (Table 2) by employing linear regression models. For the young participants, clustering coefficient and local efficiency measured at left middle temporal gyrus were negatively associated with BMI. Similar observation was obtained in (Chen et al., 2018) which linked lower structural network segregation to obesity (higher BMI). Our findings suggested that there was a mapping between brain network segregation attributes and human physical conditions, and segregation features of the left middle temporal gyrus showed their potential as neuroimaging biomarkers to detect BMI-associated complex diseases such as dementias (Emmerzaal et al., 2015), cardiovascular disease, cancer, respiratory disease and diabetes (Stenholm et al., 2017). For elderly adult participants, no significant associations were identified between segregation measures and any outcomes, which suggested an interesting topic for further explorations.

Multiple regression analyses demonstrated that middle temporal gyrus was linked to weight-related issues. For example, Veit et al. (2014) and Gómez-Apo et al. (2018) revealed that BMI, visceral fat and age were negatively associated with cortical thickness of the left middle temporal gyrus, Ou et al. (2015) indicated that greater adiposity was associated with lower gray matter (GM) volumes in the middle temporal gyrus, Yokum et al. (2012) found positive correlation between BMI and white matter (WM) volume in the middle temporal gyrus, Rapuano et al. (2016) illustrated left middle temporal gyrus was detected significantly greater activation in response to food commercials than to non-food commercials, Salzwedel et al. (2019) concluded that maternal adiposity influenced neonatal brain functional connectivity in middle temporal gyrus, and Peven et al. (2019) identified that cardiorespiratory fitness was negatively associated with functional connectivity in the right middle temporal gyrus. To the best of our knowledge, our investigations for the association between structural connectivity in the middle temporal gyrus and BMI was among the first weight-related studies with high-level imaging features measured from structural network connectivity, and our results confirmed several previous findings analyzed from thickness data, T1-weighted MRI data, and fMRI data.

For the third aspect, since there was an emerging interest in understanding the segregation and the integration of brain networks (Cohen and D’Esposito, 2016; Mohr et al., 2016) as well as other important network attributes such as centrality (Zuo et al., 2012) and resilience (Karwowski et al., 2019), our goal was to expand our focus on comprehensively discussed segregation attributes to a more complete set of network attributes including segregation, integration, centrality and resilience. For both node level network attributes measured at left and right middle temporal gyrus and global network attributes, we applied targeted genetic association analyses on global efficiency and density (integration, network level), betweeness and eigenvector centrality (centrality, node level) and assortativity coefficient (resilience, network level) of the structural connectivity. We identified several pairwise associations between rs7937515 and these network attributes in both HCP and ADNI datasets (Table 3), and noticed a significant association between rs7937515 and global efficiency in both datasets, which suggested that rs7937515 was involved into the dynamic fluctuations of segregation and integration of neural information. This finding partially answered an elusive question of revealing genetic basis for brain mechanisms of balancing network segregation and integration. Another worth noting finding was that rs7937515 was associationed density and eigenvector centrality respectively in our targeted analyses, while such associations were vanished in elderly participants, which suggested inconsistent genetic influences across different life stages.

With the awareness of the hemispheric asymmetry of network organization, a genetic basis to explain the differences in connectome between two hemispheres were under discovered. In this work, we identified an obvious inconsistency of genetic influences on human connectome in different brain hemispheres (Table 3). As reported in several recent studies (Tian et al., 2011; Shu et al., 2015; Jiang et al., 2019), the topological organizations of structural networks were not uniformly affected across brain hemispheres, which lead to a non-uniformly distributed destruction on neural network of the left and right hemispheres. Our finding gave an explanation from the point-view of genetics, with the potential for further investigations as many of the destruction on neural network (as iQT) were linked to cognitive and behavioral functions and dysfunctions, and their genetic mechanisms were still under discovered.

## 5 Conclusion

In this work, we constructed the structural network connectivity, extracted complex-network attributes and examined the heritability of network segregation measures. Then, we revealed a novel association between the minor allele (G) of rs7937515 and decreased network segregation measures of the left middle temporal gyrus across HCP young participants and ADNI elderly participants, which demonstrated a consistent genetic risk effect on brain network connectivity across lifespan. We elucidated the neurobiological pathway from SNP rs7937515 and genes *FAM86C1*/*FOLR3* to brain network segregation, and to BMI. In such pathway, we concluded a genetic risk effect on BMI due to their positive association, examined the mediated effect of network segregation measures, and discovered network segregation of the left middle temporal gyrus as BMI-related neuroimaging biomarkers by identifying a negative association between them. We also examined genetic associations of a more complete set of important network attributes including integration, centrality and resilience, and demonstrated the consistency and discrepancy in genetic associations in brain aging. At last, we illustrated hemispheric asymmetry of network organization in both datasets in the aspect of genetic effect.

In sum, with the awareness that BMI is directly and indirectly associated to multiple complex diseases, this study performed a systematic analysis that integrated genetics, connectomics and phenotypic outcome data, with the goal of revealing biological mechanisms from the genetic determinant to intermediate brain connectomic traits and to the BMI phenotype at two different life stages. We identified the genetic effect of rs7937515 on human brain network segregation in both young and elderly participants and on BMI in young adult cohort. Our findings confirmed several previous genetic and imaging biomarker discoveries. Moreover, we provided outcome-relevant genetic insights in the aspect of complex-network attributes of human brain connectome. Similar analytical strategies can be applied to future integrative studies linking genomics with connectomics, including the analyses of structural and functional connectivity measures, additional network attributes, longitudinal or dynamic connectomic features, as well as other types of brain imaging genomic data.

## 6 The Alzheimer’s Disease Neuroimaging Initiative

Data used in preparation of this article were obtained from the Alzheimer’s Disease Neuroimaging Initiative (ADNI) database (adni.loni.ucla.edu). As such, the investigators within the ADNI contributed to the design and implementation of ADNI and/or provided data, but did not participate in analysis or writing of this report. A complete listing of ADNI investigators can be found at: http://adni.loni.usc.edu.

## Data Availability

Publicly available datasets were analyzed in this study. This data can be found here: the ADNI website (http://adni.loni.usc.edu/) and the HCP website (https://www.humanconnectome.org/).
